# Long-term functional outcome in adults with severe TBI: a meta-analysis

**DOI:** 10.1186/cc12272

**Published:** 2013-03-19

**Authors:** M Asselin, Y Lachance, G Lalonde, A Boutin, M Shemilt, L Moore, F Lauzier, R Zarychanski, P Archambault, F Lamontagne, D Fergusson, A Turgeon

**Affiliations:** 1 Université Laval, Québec, Canada; 2 University of Manitoba, Winnipeg, Canada; 3 University of Ottawa, Canada

## Introduction

Severe traumatic brain injury (TBI) is a major cause of death and of severe neurologic sequelae. Long-term functional outcome of TBI and its best timing of assessment are not well understood, and may be evaluated too prematurely in clinical studies because of resources required to do so without too much missing data. Hence, we conducted a systematic review of studies in severe TBI patients to evaluate the long-term functional outcome. We hypothesized that functional impact measured by the Glasgow Outcome Scale (GOS), or the extended version (GOSe), may plateau after several months in patients with severe TBI.

## Methods

We performed a systematic review of randomized controlled trials and cohort studies (prospective and retrospective) in patients with severe TBI. We searched MEDLINE, Embase, Cochrane Central, BIOSIS, CINAHL and Trip Database from their inception to December 2011. References of included studies were searched for additional studies. Two reviewers independently determined study eligibility and collected data. The primary outcome measure was the proportion of unfavourable functional outcome (GOS 1 to 3 or GOSe 1 to 4) at 6 to 12 months, 12 to 18 months, 18 to 24 months and more than 24 months after severe TBI. We calculated Freeman Tukey-type arcsine square-root transformations and pooled data using random-effect models. Heterogeneity was assessed with the *I*^2 ^test and sensitivity analyses were based on *a priori *hypotheses.

## Results

In total, 4,432 studies were assessed for eligibility; 209 studies (*n = *31,540) were included. In the 188 studies using the GOS, a poor functional outcome was observed in 56.6% (95% CI = 54.0 to 59.1%, *I*^2 ^= 91%), 51.9% (95% CI = 38.0 to 59.0%, *I*^2 ^= 84%), 57.0% (95% CI = 48.2 to 55.5%, *I*^2 ^= 93%) and 56.9% (95% CI = 48.2 to 65.1%, *I*^2 ^= 93%) of patients at 6 to 12 months, 12 to 18 months, 18 to 24 months and beyond 24 months, respectively. In the 18 studies using GOSe, a poor functional outcome was observed in 62.9% of patients at 6 to 12 months (95% CI = 55.9 to 69.2%, *I*^2 ^= 90%) and 54.6% at 12 to 18 months (95% CI = 43.2 to 65.8%, *I*^2 ^= 90%). Heterogeneity was present in most analyses and was not entirely explained by the planned sensitivity analyses.

## Conclusion

Considering that the incidence of patients with an unfavourable outcome remained constant at different assessments, a follow-up of severe TBI patients longer than 12 months does not provide incremental information. Functional outcomes measured longer than 12 months after the injury may not be warranted in clinical studies.

